# The burden of primary headache disorders in Zambia: national estimates from a population-based door-to-door survey

**DOI:** 10.1186/s10194-015-0513-9

**Published:** 2015-04-29

**Authors:** Edward Mbewe, Pachuau Zairemthiama, Ravi Paul, Gretchen L Birbeck, Timothy J Steiner

**Affiliations:** Chainama College of Health Sciences, Lusaka, Zambia; Chikankata Hospital, Mazabuka, Zambia; Department of Psychiatry, School of Medicine, University of Zambia, Lusaka, Zambia; Department of Neurology, University of Rochester, Rochester, NY USA; Department of Medicine, University of Zambia, Lusaka, Zambia; Department of Neuroscience, Norwegian University of Science and Technology, Edvard Griegs Gate, NO-7491 Trondheim, Norway; Division of Brain Sciences, Imperial College London, London, UK

**Keywords:** Epidemiology, Burden of disease, Headache, Migraine, Tension-type headache, Medication-overuse headache, Sub-Saharan Africa, Global Campaign against Headache

## Abstract

**Background:**

Three headache disorders – migraine, tension-type headache (TTH) and medication-overuse headache (MOH) – are major contributors to population ill-health. Policy-makers need local knowledge of these to guide priority-setting. Earlier we reported the prevalence of these disorders in Zambia; here we describe the burdens attributable to them.

**Methods:**

This was a cross-sectional population-based survey of adults aged 18-65 years, selected by cluster-randomized sampling in the mostly urban Lusaka Province and mostly rural Southern Province. Interviewers visiting households used a structured questionnaire. Diagnoses made algorithmically applied ICHD-II criteria. Burden enquiry focused on the previous 3 months and the day before interview. Disability was estimated by applying disability weights (DWs) from the Global Burden of Disease Survey 2010.

**Results:**

From 1,134 households, 1,085 unrelated adults (450 male, 635 female) were interviewed (refusal rate 4.3%). The gender- and habitation-adjusted 1-year prevalence of migraine was 22.9%, of TTH 22.8%, of headache on ≥15 days/month 11.5%, of probable MOH (pMOH) 7.1%. Reported mean intensity of migraine attacks was 2.7, representing severe pain. People with migraine spent 10.0% of their time in the ictal state (DW: 0.433); they were therefore 4.3% disabled overall. Disability from TTH was much lower. People with pMOH (time with headache: 37.5%; DW: 0.220) were 8.3% disabled overall. Average lost productive time in the preceding 3 months for migraine was 4.1 days from work (6.3% loss) and 4.2 days (4.7% loss) from household work. Losses for pMOH were 4.8 days (7.4% loss) from work and 4.5 days (5.0% loss) from household work. In the population aged 18-65 years (effectively the working population), estimated disability from migraine was 0.98%, with 1.4% of workdays lost, and from pMOH was 0.59%, with 0.53% of workdays lost. Headache yesterday was reported by 28.3% of participants, whose average productivity yesterday was 55.9% of expectation.

**Conclusions:**

Zambia loses 1.93% of GDP to headache, and action is required to mitigate this loss and the associated suffering. Structured headache services with their basis in primary care are the most efficient, effective, affordable and equitable solution. They could be implemented within the existing health-care infrastructure of Zambia. These matters require urgent political attention.

## Background

Three headache disorders – migraine, tension-type headache (TTH) and medication-overuse headache (MOH) – are major contributors to population ill-health. Worldwide, migraine is the seventh leading specific cause of years of life lost to disability (YLDs), responsible for 2.9% of all YLDs, and more than half of all YLDs attributable to neurological disorders [1-3]. This represents a substantial burden of disease. TTH is more prevalent than migraine [2], but less disabling at an individual level. In the Global Burden of Disease Survey 2010 (GBD2010), it did not make a substantial additional contribution to YLDs attributable to headache [1,2]. Both migraine and TTH are primary headache disorders, but both can lead, through mistreatment, to MOH. This secondary disorder by definition occurs on ≥15 days/month, and is a major contributor to disability burden at individual level [4]. Almost certainly it is at population level also [5,6].

For health policy-makers, all of this is important knowledge. Not only do headache disorders affect very large numbers of people through their high prevalence [1,2], imposing symptom burdens of pain, suffering and impaired quality of life, but through this disability burden they cause productivity losses that carry a very substantial financial penalty [4]. Although health-care solutions may be challenging to implement for such large numbers of people, it is neither humane nor economically sensible to ignore the public-health imperative of providing care for people with headache [7]. Effective treatments do exist [8] and, appropriately used, are likely to be cost-saving in most economies [7].

At national levels, policy-makers need *local* knowledge to guide decision-making and priority-setting. For headache disorders, this has been unavailable in many countries of the world [9]. Following its launch in 2003, the Global Campaign against Headache [10] set out to fill the major knowledge gaps as its first objective [11,12], developing standard methodology and survey instruments for population-based studies [13,14]. In sub-Saharan Africa (SSA), there had been few studies of prevalence or burden of primary headache disorders, and most of these had been in select sub-populations rather than population-based, with widely varying prevalence findings [15-23] but all consistent in one respect: they were considerably lower than global averages [9]. We undertook a population-based study in Zambia, as a project within the Global Campaign against Headache [10], expressly to inform health policy-makers [24]. It was the first such study in the African Region. It revealed that the prevalences of headache generally and of migraine specifically were in line with those observed in Global Campaign studies conducted with similar methodology elsewhere: in other words, headache is as common in SSA as in higher-income countries. Furthermore, we noted a particular problem in urban Zambia of headache on ≥15 days/month, much of it probable MOH (pMOH), which was far more prevalent there than in any other country studied [24].

Here we report the burdens attributable to these prevalent disorders. We focus on symptom burden, disability and lost productive time. In addition, a population-based study in Georgia, another lower-middle income country [25], established that people there with headache were willing to pay for treatment at a level that might significantly complement public services [26]. Knowledge of willingness-to-pay (WTP) for health care in the context of ability to pay in resource-limited African settings might help direct health-policy, priority-setting and decision-making; therefore we also enquired into WTP.

## Methods

### Ethics

This study was approved by the University of Zambia’s Research Ethics Committee. Verbal informed consent was obtained from key informants and all participants before we asked the survey questions.

### Study design and procedures

The design has been described in full previously [24] and is summarised here. The study was a cross-sectional population-based survey of adults aged 18-65 years, selected by cluster-randomized sampling in the mostly urban Lusaka Province and the mostly rural Southern Province. In each Province, interviewers randomly selected blocks or circumscribed collections of dwellings (clusters), one or more dwellings within each of these, and one adult participant within each household’s family.

In Lusaka Province, interviewers were interested faculty and advanced students from the Chainama College of Health Sciences. In the Southern Province, interviews were conducted by the Chikankata Epilepsy Care team, whose staff had been conducting community and hospital-based health-related research for over a decade. All received training for this study.

Interviewers used a local culturally-adapted version of the structured questionnaire developed for these surveys by *Lifting The Burden* (LTB) [27], and used previously in Russia [28], China [29] and India [30] and subsequently in a total of 19 countries in 18 languages [14]. This was translated according to LTB’s translation protocol [31] from English into three local languages: Bemba and Nyanja for Lusaka Province, and Tonga for the Southern Province.

Enquiry was into lifetime, 1-year and 1-day prevalence of headache and 1-year prevalence migraine, TTH, headache on ≥15 days/month and pMOH. Participants who identified more than one headache type were asked to focus only on the one most bothersome to them, so diagnoses were mutually exclusive. Interviewers did not make diagnoses. These were derived during analysis, algorithmically [14], from the recorded survey responses. Participants reporting headache on ≥15 days/month were first separated from this process, and described as a separate group because they cannot be adequately diagnosed by questionnaire [14]. However, those also reporting use of headache medication regularly on >3 days/week were considered to have pMOH. To all others, the algorithm applied ICHD-II criteria in the order: migraine, TTH, probable migraine, probable TTH [32]. Cases of migraine and probable migraine, and of TTH and probable TTH, were then combined for prevalence estimation and further analyses [13]. The remaining cases were unclassified.

Burden enquiry focused on the previous 3 months and, in those to whom it was relevant, on the day before the interview. The latter information did not depend upon the participant’s long-term memory, but the interview was not structured to diagnose this headache if the participant had more than one headache type. Symptom burden was expressed in terms of frequency, intensity and duration. Questions were also asked about headache-related lost productivity at work and in the household using the Headache-Attributed Lost Time (HALT) questionnaire [33], and WTP.

### Analysis and statistics

We recorded typical headache intensity on a verbal rating scale (“not bad”, “quite bad” and “very bad”), transformed these data into a numerical scale 1-3, and treated them as continuous data, calculating a mean for the sample. We recorded headache frequency as continuous data in days affected per month. We recorded duration of headache as continuous data in hours. For both these we calculated means for the sample, and from them derived the average time spent in the ictal state. To calculate disability we used disability weights (DWs) from GBD2010 [34].

All analyses were performed with SAS version 9.2 (SAS Institute Inc., Cary, NC, USA) or Excel 2007 (Microsoft Corporation, Redmond, WA, USA). Statistics are mainly descriptive. When appropriate we calculated *P*-values as an aid to interpretation, using Student’s *t* test and chi-squared to compare distributions and proportions.

## Results

Of 1,134 unrelated household members aged 18-65 years with whom contact was made, 1,085 (450 male, 635 female; 198 rural [Southern Province], 887 urban [Lusaka Province]) consented to be interviewed (refusal rate 4.3%). The male/female ratio (41.5:58.5) diverged from the national ratio for this age range (very close to 50:50) [25]; the ratio of urban/rural dwelling (82:18) also did not match the urban/rural distribution (40:60) of the Zambian population [25]. Statistical adjustments to observed prevalences were therefore necessary for both gender and habitation [24]. In other respects our samples were demographically comparable to the Lusaka and Southern Province general populations [24,25].

The prevalence data have been reported previously [24]. In summary, 781 participants (72.0%; males 66.2%, females 76.1%) reported headache unrelated to another illness in the past year, and 307 (28.3%; males 21.3%, females 33.1%) reported headache on the day prior to the interview (headache yesterday). Adjusted for gender and habitation, the 1-year prevalence of any headache was 61.6% and the point prevalence (headache yesterday) was 19.1%. The gender- and habitation-adjusted 1-year prevalence of migraine was 22.9%, of TTH 22.8%, of headache on ≥15 days/month 11.5%, of pMOH 7.1%. Prevalence of pMOH was much higher in urban (14.5% gender-adjusted) than in rural populations (2.1%).

### Individual burden

The individual symptom burden arising from migraine is shown in Table 1. Reported mean intensity of attacks was 2.7, equating to severe pain. Headache was reported on an average of 3.4 days/month [24], with a mean duration of 36.4 hours. As this was >24 hours, it implied a mean attack frequency of 2/month, each extending into 2 days. The consequence would be 10.0% of all time spent in the ictal state (calculated as 100*[2*36.4*12]/[365*24]).

**Table 1:** **Symptom and disability burdens arising from migraine at individual and population levels**

**Burden variable**	**Value**
Mean intensity of attack (on scale 0-3)	2.7
Mean attack frequency (per month)	3.4
Mean duration of attack (hr)	36.4
Mean time in ictal state (% of total time)	10.0
Disability weight (from GBD2010)	0.433
Mean disability per person with migraine (%)	4.3
Prevalence (adults aged 18-65 yr) (%)	22.9
Disability in entire 18-65 yr-old population (%)	0.98

Applying the GBD2010 DW of 0.433 for the ictal state of migraine [34] generated a disability level of 4.3% (0.433*10.0%). This was the mean estimated disability attributed to migraine per adult with the disorder in Zambia.

We did not complete similar estimates for TTH. Headache intensity for TTH was 1.9 (moderate), with a mean of 2.5 days/month recorded with headache. The GBD2010 DW for the ictal state of TTH was only 0.04 [34], so the mean estimated disability attributed to TTH per adult with the disorder would be quite low.

For pMOH, we did not have reliable reported data for time spent in the ictal state. Most participants claimed to have headache every day (mean 29.8 days/month), and by definition it occurs on ≥15 days/month. We took a more conservative estimate of 75%, and assumed headache was present for only one half of the time because of the effect of treatment. Thus we calculated mean time in ictal state as 37.5% of total time. GBD2010 did not report a DW for MOH, but this disorder was included in the global consultation for deriving DWs: for the headache state of MOH, a DW of 0.220 was assigned [unpublished]. Thus the mean estimated disability attributed to MOH per adult with this disorder would be 8.3% (0.220*37.5%).

We had data for lost productive time to relate to these estimates, deriving from the HALT questionnaire [33] (Table 2). On average, people with migraine lost 4.1 days from work in the preceding 3 months, and much the same, 4.2 days, from household work. Assuming a 13-week period had 65 workdays, the former represented a 6.3% loss, compared with the estimated disability level of 4.3%. For household work, the denominator was presumably 90 days, in which case the loss was 4.7%. The quartiles make clear that a highly-disabled minority accounted for a substantial proportion of these losses. The losses for people with pMOH were somewhat but not very much greater: from paid work, 4.8 days represented a 7.4% loss, and from household work 4.5% represented 5.0% compared with the estimated disability level of 8.3%. Again, a highly disabled minority accounted for much of these losses. More than half of people with TTH lost no productive time at all, and total losses to this disorder were low.

**Table 2:** **Lost productive time in preceding 3 months according to HALT questionnaire**

**Diagnosis**	**Days lost from paid work**	**Days lost from household work**	**Days lost from leisure**
Migraine	4.1 (6.6)	4.2 (7.4)	1.3 (2.8)
	[0; 2; 5; 45]	[0, 2, 5, 67]	[0; 1; 0; 30]
TTH	1.4 (2.8)	1.1 (3.0)	0.4 (1.2)
	[0; 0; 2; 24]	[0; 0; 1; 35]	[0; 0; 0; 14]
pMOH	4.8 (8.2)	4.5 (7.3)	1.0 (1.8)
	[0; 3; 7; 60]	[0; 4; 6; 70]	[0; 1; 1; 15]

Values are means (SD) [quartile 1; median; quartile 3; upper limit of range].

TTH: tension-type headache; pMOH: probable medication-overuse headache.

We also had data on WTP for effective health care for headache, if it were available, and on income to set this in context (Table 3). Both questions were answered fully by 651 participants. Income levels in Zambia were low – around USD 200 per month on average, with a modest difference between migraine and TTH (*P* = 0.07) but those with pMOH had significantly lower income (2-tailed Student’s *t* test *vs* migraine: *P* < 0.0001). WTP perhaps reflected this: means ranged from USD 1.54 to USD 1.67 per month, or, as a percentage of income, from 1.4 to 1.8%. Differences between the diagnoses in absolute WTP were small and insignificant; in percentage terms, those with migraine were willing to pay less than all others, but this appears artefactual. A total of 39 participants claimed a WTP >5% of their income, 14 (5.6%) diagnosed with migraine (maximally 33%), 15 (5.7%) with TTH (maximally 50%) and 10 (7.3%) with pMOH (maximally 25%).

**Table 3:** **Willingness to pay for effective care, against diagnosis and income**

**Burden variable**	**Migraine (n = 252)**	**TTH (n = 262)**	**pMOH (n = 137)**
Income per month (USD) (mean [SD])	240 [218]	206 [190]	158 [160]
Willingness to pay (USD) (mean [SD])	1.67 [2.85]	1.60 [2.80]	1.54 [2.62]
% WTP of income (mean [SD])	1.4% [3.0]	1.8% [5.1]	1.8% [3.5]

TTH: tension-type headache; pMOH: probable medication-overuse headache.

### Population-level burden

Returning to Tables 1 and 2, we made estimates of burden at population level, and on society. Each person with migraine was 4.3% disabled, while the prevalence of migraine among 18-65 year-olds was 22.9%. Thus there was a 0.98% (4.3*0.229) disability attributable to migraine among the population of this age. Those with migraine lost 6.3% of workdays, which diluted to 1.4% among this population, somewhat greater than the estimated underlying disability.

Similar estimates were possible and informative for pMOH. Individual disability was 8.3%; prevalence 7.1%. The calculated population-level disability from pMOH was 0.59%%. Lost workdays were 7.4%, and therefore 0.53% among the population of 18-65 year-olds, only slightly less than the conservatively estimated underlying disability.

### Headache yesterday

In our earlier manuscript, we noted the high headache frequency overall of 10.3 days/month, creating, among the 72.0% with headache, a probability of headache on any particular day of 0.34 [24]. The predicted 1-day prevalence of headache was therefore 24.5%, slightly less but consistent with the 28.3% prevalence of headache yesterday reported in the sample. We also enquired into effect of headache yesterday on activities yesterday. Of 307 responders, only 77 (25.1%) reported that they were able to do everything as normal and a further 45 (14.7%) that they could do more than half of their planned activities; much larger numbers claimed they could do less than half (117; 38.1%) or nothing at all (115; 37.5%). We interpreted these reports conservatively, assuming the intermediate groups actually managed 80% and 50% respectively, and that the “could-do-nothing” group probably achieved 25%. On this basis, the average “output” per person with headache yesterday was 55.9% of expectation. The deficit, spread among the population according to the gender- and habitation-adjusted prevalence of 19.1%, would be 10.7%, considerably higher than other population estimates of burden.

## Discussion

Just as our first manuscript showed headache to be highly prevalent in Zambia – no less so than elsewhere in the world [24] – here we have demonstrated that there are high levels of burden arising from it. Symptom burdens affect individuals, particularly those with migraine (22.9% of the adult population) or pMOH (7.1%). The former spend, on average, an estimated 10.0% of all their time in the ictal state, which is a very substantial loss of healthy time, with headache described as severe (2.7 on a scale 0-3). The consequent disability burden averaged over time is 4.3%. As might be expected, people with pMOH carry much more individual disability – estimated at 8.3%.

These estimates were not exactly reflected in other measures of burden. People with migraine lost 6.3% of workdays and 4.7% of household workdays; more than might be expected in the case of the former, but the discrepancy might be explained by the very skewed distribution and a highly-disabled minority. What is at first surprising is that losses from people with pMOH were only 7.4% from workdays and 5.0% from household workdays – higher than for migraine but not greatly so, whereas the disability estimate was two-fold higher. The explanation almost certainly lies in the disruptive effect of episodic migraine, with its short sharp bursts of high disability, which makes coping difficult. People manage chronic pain and disability differently, with more emphasis on distraction and less on palliation [35]. Nonetheless, the greater disability among people with MOH was, perhaps, reflected in their lower incomes despite that they were predominantly urban-dwellers.

Perhaps this was why it was migraine, not pMOH, that generated the highest WTP in absolute terms; indeed, pMOH generated the lowest. Of course, other factors come into play with WTP, including affordability, and, set against income, people with migraine (who had the highest incomes) demonstrated the lowest relative WTP. It should be noted that none of these differences was significant. Respondents with pMOH must already be spending significant amounts on medication, presumably with perceived benefit, which would condition their responses to the enquiry. This does question their low WTP, unless they saw the enquiry as asking what *more* they would pay than they were already. The 39 respondents who would pay >5% of their income (up to 50%, although they might not pay this in reality) were distributed reasonably equally between the three diagnostic groups, slightly more having pMOH.

Part of our purpose was to inform policy, which the population-level findings do very well. Individual disability of 4.3% attributable to migraine extrapolated to a disability of 0.98% among the entire population aged 18-65 years – effectively the working population. It is important to recognise this. Individual disability of 8.3% among those with pMOH extrapolated to 0.59% in the population. The total was 1.6%, not including TTH (which added little). Lost workdays from migraine (6.3%) and pMOH (7.4%) translated to 1.4% and 0.53% respectively, which together represented a loss of workforce capacity of 1.93%, and this huge economic burden would be reflected in national productivity and gross domestic product (GDP).

We would like to make comparisons with other countries, but there is no point in doing so when methodological differences may be the explanation of any differences [13]. Therefore we limit our observations to LTB studies, and focus on Georgia and India, both lower-middle income countries like Zambia [25]. LTB has completed studies in Nepal, Pakistan and Ethiopia, but these are not yet fully analysed for publication. In Georgia, with a much lower 1-year prevalence of 13.7% [36], people with migraine lost 5.7% of workdays (quite similar to Zambia) and 4.4% of household workdays (almost the same) [26]. Losses for people with headache on ≥15 days/month (prevalence 7.6% [36], two thirds that of Zambia) were 6.8% from workdays and 5.5% from household workdays [26], very similar to those from pMOH in Zambia. In other words, while prevalences differed between these two countries of both migraine and headache on ≥15 days/month, *recalled* lost productivity per person over 3 months was much the same (the emphasis here being deliberate, as will be seen later).

In Georgia, WTP was several-fold higher in absolute terms (USD 8 per month for 93% of respondents to the questions), and sufficient in most cases to pay for effective care [26]. In Zambia, WTP would contribute little towards the cost of care. In relative terms, set against GDP (USD 16.13 bn for Georgia *versus* Zambia’s USD 22.38 bn [25]), the disparity was even greater. As in Zambia, WTP in Georgia did not correlate with headache type or frequency, or with lost productive time, which means the explanation for the different levels of WTP is probably cultural. In Georgia, there is no expectation of free health care, so the concept of paying for care is not unusual.

From India, with a 1-year migraine prevalence of 25.6% [unpublished], slightly higher than that in Zambia, but with nowhere near the level of headache on ≥15 days/month, we have comparative lost workforce capacity data: for all headache, 1.1% [37]. In Zambia more than this loss is suffered from migraine alone, with pMOH adding half as much. The estimate from India is, as elsewhere, based on participants’ recall of the preceding 3 months, but it was very closely corroborated by those reporting headache and consequential productivity losses on the day before their interview. Which brings us to an obvious discrepancy in the headache yesterday data from Zambia.

Enquiry into headache yesterday obviates the problem of faulty recall – highly likely over periods of 3 months [13]. In fact, based on reported frequency over the preceding 3 months, the predicted 1-day prevalence of headache was 24.5%, which reassuringly matched the 28.3% reported prevalence of headache yesterday (the difference being the numerical part of the recall error). But when it came to effect of headache on activities yesterday, even with conservative interpretation, average “output” per person with headache yesterday was 55.9% of what had been intended. The deficit, spread among the population, would be 10.7%, considerably higher than other population estimates, and actually unfeasible as an estimate of headache-attributed population disability assumed to be present every day. This phenomenon has not been encountered in the few surveys of headache yesterday so far conducted in other countries [37-39], and any explanation is speculative. One is that, thinking of yesterday, attention focused on and gave undue weight to many small things left undone. Another is that, unexpectedly having the undivided attention of a health-care worker on the doorstep the day after a headache episode, a participant did not wish to downplay its effect in any way. These are not unlikely explanations, and we must accept that an unknown degree of exaggeration inflated these accounts of productivity loss yesterday. The much lower estimates based on recall over the preceding three months are similar to those from Georgia, also based on recall. The truth in Zambia lies somewhere between the two estimates of population disability and productivity loss.

It is a study limitation that we cannot be precise about this. In our earlier paper, we also recorded our inability to complete the diagnostic validation exercise satisfactorily owing to country-based factors beyond our control [24], but the essential messages here, relating to headache and medication overuse, are not significantly affected by this limitation. The study had several strengths, also noted previously [24]. We employed population-based sampling, included diverse regions with a sample size of >1,000, and used ICHD-II diagnostic criteria [32]. The methodology was established [13], having been tested in numerous other countries.

## Conclusions: what is to be done?

Headache disorders are not only common in Zambia but also highly burdensome. However, burden does not sit quite as might be expected: measures of disability and lost productivity show only loose relationships with headache type and frequency.

At population level, Zambia effectively loses 1.93% of its GDP to headache – more than in Georgia [26] and almost double the loss in India [37]. Zambia also loses more of its GDP to headache than Russia (1.75% [40]) and China (1.9% [41]), other countries in which LTB has made estimates by the same methods.

Underlying these losses are the high prevalences of headache disorders, especially pMOH. These are treatable disorders [8]. Much could – and should – be done to alleviate them, partly with the expectation of substantial cost-saving [7] but also because people with headache lose much of their quality of life. Structured headache services with their basis in primary care are the most efficient, effective, affordable and equitable solution [7]; the model proposed for Europe [42] is highly adaptable, and could be reworked to match the health-care infrastructure of Zambia. These matters require urgent political attention.

Inaction is not an option: medication overuse, and MOH, are predictable consequences of lack of health care for migraine and TTH (especially when these are themselves prevalent), lack of public health education, and easy access to non-prescription analgesics (hence the urban-rural divide for pMOH: 14.5% *vs* 2.1%). In other words, a remediable problem will instead become worse.

## Abbreviations

DW: Disability weight
GBD: Global Burden of Disease
GDP: Gross domestic product
ICHD: International Classification of Headache Disorders
HALT: Headache-attributed lost time
LTB: *Lifting The Burden*
MOH: Medication-overuse headache
pMOH: Probable MOH
SSA: Sub-Saharan Africa
TTH: Tension-type headache
WHO: World Health Organization
YLD: Year of life lost to disability
